# Direct Activity Measurement of Heterotrimeric Gi Proteins and Gq Protein By Effector Pulldown

**DOI:** 10.21769/BioProtoc.5406

**Published:** 2025-08-05

**Authors:** Keiichiro Tanaka, Martin A. Schwartz

**Affiliations:** 1 Yale Cardiovascular Research Center, Section of Cardiovascular Medicine, Department of Internal Medicine, School of Medicine, Yale University, New Haven, CT, USA; 2 Department of Cell Biology, Yale University, New Haven, CT, USA; 3 Department of Biomedical Engineering, Yale University, New Haven, CT, USA

**Keywords:** GPCR, Heterotrimeric Gα protein, Gi, Gq, GINIP, GRK2, Affinity pull-down, GTPase activity

## Abstract

Studying G protein-coupled receptor (GPCR) activation of heterotrimeric G proteins is crucial for understanding diverse physiological processes and developing novel therapeutics. Traditional methods to assay GPCR activation of G proteins, including assays of second messengers and biosensors, involve complex or indirect procedures. However, second messengers like cAMP and calcium are not direct readouts of GPCR activity due to signaling crosstalk, while biosensors can have undesired consequences due to structural alteration caused by fluorescent protein insertion. Here, we present a streamlined protocol employing GST-tagged bait proteins and epitope-embedded Gα subunits to achieve direct monitoring of Gα activity within cells. This method involves purification of GST-tagged bait constructs from bacteria and subsequent direct interaction studies with GluGlu-tagged Gα proteins expressed in any human cells of interest by including GST-tagged bait proteins in the cell lysis buffer. The approach enables sensitive detection of activated Gα within cells following extracellular stimulation. Advantages of this protocol include high sensitivity, enhanced monitoring of GPCR signaling dynamics under physiologically relevant conditions with minimum alteration in Gα, and the ability to distinguish between highly homologous isoforms within the same Gα family.

Key features

• Improved analysis of GPCR-Gα signaling: Establishes a novel effector pulldown method to monitor Gα activation within cells.

• Endogenous GPCR activity measurement with increased sensitivity: Enables assays of endogenous physiological GPCR activities with improved sensitivity by increasing sample sizes.

• Epitope Incorporation without affecting functions: Uses epitope tag sequence with a few amino acid substitutions functioning like wild-type proteins, adaptable for any endogenous Gα assay if suitable antibodies are available.

• Isoform distinction: Distinguishes Gα isoforms by using embedded epitope.

## Graphical overview


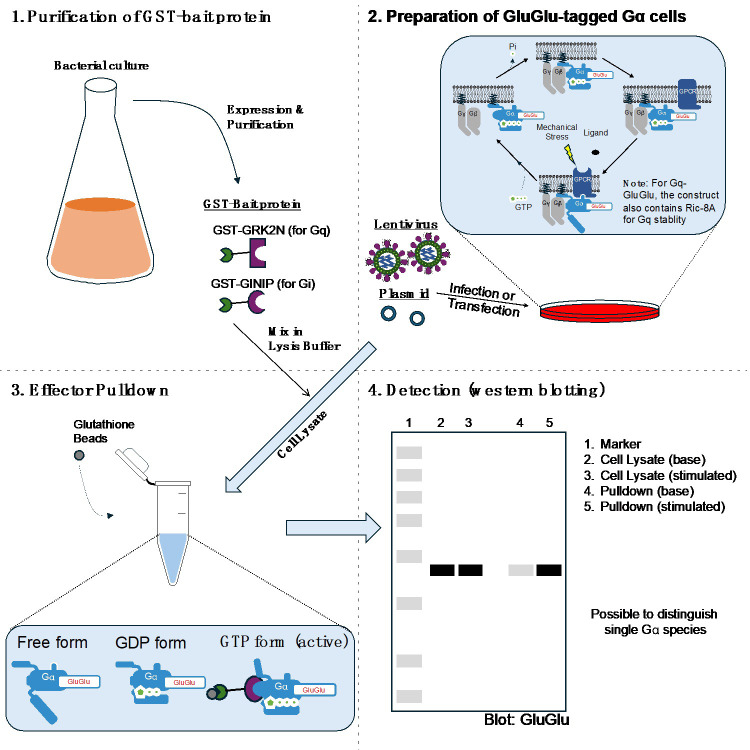


**Four major steps in the effector pull-down assay to measure Gα activity within cells.** (1) Purification of GST-bait protein, (2) preparation of GluGlu-tagged Gα cells, (3) effector pulldown, and (4) detection by western blotting.

## Background

G-protein coupled receptors (GPCRs) comprise a large family of membrane proteins that mediate responses to a vast range of extracellular signals. These include neurotransmitters, hormones, metabolites, and photons [1,2]. Consequently, they are essential for many physiological processes, and their dysregulation frequently leads to human diseases. Conversely, GPCRs are the targets of 30%–40% of all FDA-approved drugs.

GPCRs transduce extracellular stimuli through activation of heterotrimeric Gα proteins [2,3], although previous studies demonstrated that they can also initiate signaling through coupling to β-arrestins. The critical event in Gα protein activation is the exchange of guanosine diphosphate (GDP) with guanosine triphosphate (GTP), which is catalyzed by the guanine nucleotide exchange factor (GEF) activity of activated GPCRs [1]. GTP loading induces dissociation of Gα from Gβγ, both of which then activate various downstream effectors. Humans possess four major Gα protein families (Gi, Gq/11, Gs, and G12/13), each of which activates specific effectors [1]. Gα protein signaling is terminated by their intrinsic GTPase activity, which then allows reassembly of the heterotrimeric Gα-Gβγ complex. Additionally, signaling is regulated by multiple associated proteins that modulate Gα GTPase activity or conformation [4]. For instance, some Gq family proteins require chaperone proteins such as Ric-8A for their proper function [5].

Developing tools to monitor G-protein activity with high sensitivity is crucial to elucidate the complex signaling processes during GPCR to Gα activation and their feedback regulation. Measuring downstream effectors, such as cAMP and calcium, as proxies for Gα protein activation has significant caveats since their responses are not generally equivalent to GPCR-Gα signaling due to pathway crosstalk and subsequent signal amplification or suppression. Alternatively, multiple biosensors have been developed for monitoring the dissociation of Gα and Gβγ subunits [6–8]. However, these biosensors require the expression of multiple genetic components as well as significant structural alteration in Gα by embedding relatively large fluorescent proteins or luciferase, which have potentially undesired consequences. Additionally, Gα isoforms (even in the same Gα family) can specifically couple to distinct receptors [9] and regulate specific physiological processes, which biosensors do not readily distinguish.

**Figure 1.**
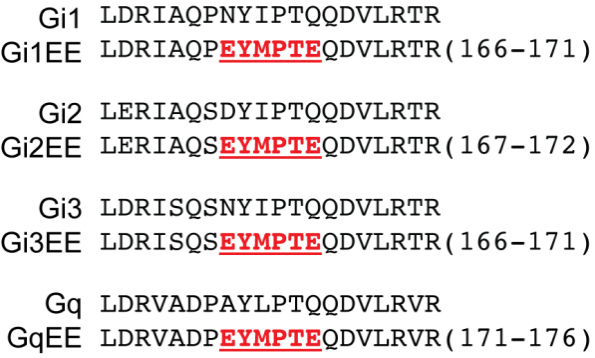
GluGlu-tag embedded Gα protein sequence. Red highlights a GluGlu epitope sequence. GluGlu epitope is derived from a 314–319 amino acid sequence of the middle T antigen of mouse polyoma virus [15]. Sequences are obtained from Tanaka et al. [16].

To address these challenges, we employed pull-down assays to directly detect Gα proteins in their activated state. To enhance sensitivity and allow isoform-specific detection, we expressed Gα subunits with inserted GluGlu epitope tags (**Figure 1**). These Gα mutants are tagged by mutation of a short stretch of amino acids to create GluGlu epitopes, without perturbing function [10,11]. Historically, GTPase effector pulldowns were developed to detect small GTPase activation [12]. It was previously reported that GINIP binds specifically to active, GTP-bound Gi [13], while the N-terminal region of GRK2 binds specifically to active Gq [14]. Consequently, we found these GluGlu-tagged Gα mutants selectively bind to GINIP or GRK2 N-terminal region when they are in the activated GTP-bound state.

Using this assay, we successfully monitored unique G protein activation by endogenous GPCRs, with the additional advantage of increasing sensitivity by collecting large lysate volumes. Refining these methodologies as described here in detail will provide more precise insights into GPCR signaling and their roles in health and disease.

## Materials and reagents

**Biological materials**

1. One Shot^TM ^Stbl3^TM^ chemically competent *E. coli* (Invitrogen, catalog number: C737303)

2. BL21(DE3) competent cells (Thermo Fisher Scientific, catalog number: EC0114)

3. Lenti-X^TM^ 293T cell line (Takara, catalog number: 632180)

4. HUVECs (human umbilical vein endothelial cells) (Yale Vascular Biology & Therapeutics Program, catalog number: T75SC-1)

**Reagents**

1. Constructs expressing GST-tagged GINIP protein (kind gift from Dr. Mozlich) [13]

2. Constructs expressing GST-tagged GRK2 N-terminal domain (Tanaka et al., Dr. Schwartz lab; File S1)

3. Constructs expressing GluGlu-epitope inserted human Gi1 protein (Tanaka et al., Dr. Schwartz lab; File S1)

4. Constructs expressing GluGlu-epitope inserted human Gi2 protein (Tanaka et al., Dr. Schwartz lab; File S1)

5. Constructs expressing GluGlu-epitope inserted human Gi3 protein (Tanaka et al., Dr. Schwartz lab; File S1)

6. Constructs expressing GluGlu-epitope inserted human Gq protein and Ric8A protein (Tanaka et al., Dr. Schwartz lab; File S1)

7. Constructs expressing GluGlu-epitope inserted human Gi1 Q204L protein (Tanaka et al., Dr. Schwartz lab; File S1)

8. Constructs expressing GluGlu-epitope inserted human Gi2 Q205L protein (Tanaka et al., Dr. Schwartz lab; File S1)

9. Constructs expressing GluGlu-epitope inserted human Gi3 Q204L protein (Tanaka et al., Dr. Schwartz lab; File S1)

10. Constructs expressing GluGlu-epitope inserted human Gq Q209L protein (Tanaka et al., Dr. Schwartz lab; File S1)

11. pcDNA5/FRT-HA-hM3D(Gq) (Addgene, catalog number: 45547)

12. pcDNA5/FRT-HA-hM4D(Gi) (Addgene, catalog number: 45548)

13. Lentivirus packaging plasmid (psPAX2) (Addgene, catalog number: 12260)

14. Lentivirus envelope plasmid (pMD2.G) (Addgene, catalog number: 12259)

15. Ampicillin sodium salt (Sigma, catalog number: A0166-25G)

16. LB medium (capsules) (MP Biomedicals, catalog number: 3002021)

17. Bacto^TM^ agar (BD, catalog number: 214010)

18. Bacto^TM^ yeast extract (Gibco, catalog number: 212750)

19. Bacto^TM^ tryptone (Gibco, catalog number: 211705)

20. Glycerol (Sigma, catalog number: G7893)

21. Sodium chloride (NaCl) (J.T. Baker, catalog number: 3624-05)

22. IPTG (isopropyl β-D-1-thiogalactopyranoside) (AmericanBio, catalog number: 18501800)

23. KH_2_PO_4_ (J.T. Baker, catalog number: 3246-05)

24. K_2_HPO_4_ (J.T. Baker, catalog number: 3252-01)

25. Trizma^®^ base (Sigma, catalog number: T6066)

26. Hydrogen chloride (HCl) (Sigma, catalog number: H1758-500ML)

27. Triton X-100 (AmericanBio, catalog number: AB02025)

28. Dithiothreitol (DTT) (Sigma, catalog number: D9779)

29. Lysozyme from chicken egg white (Sigma, catalog number: L6876-10G)

30. cOmplete^TM^, mini, EDTA-free protease inhibitor cocktail (Sigma, catalog number: 11836170001)

31. Halt^TM^ protease and phosphatase inhibitor cocktail (100×) (Thermo Fisher Scientific, catalog number: 78440)

32. Glutathione Sepharose 4B (Millipore/Sigma, catalog number: 17-0756-01)

33. Sodium hydroxide (NaOH) (J.T. Baker, catalog number: 3115-05)

34. L-Glutathione reduced (Sigma, catalog number: G6529-1G)

35. Bio-Rad Protein Assay Dye Reagent Concentrate (Bio-Rad, catalog number: 5000006)

36. DMEM, high glucose, pyruvate (Gibco, catalog number: 11995073)

37. Fetal bovine serum (FBS) (Phoenix-Scientific, catalog number: PS-100-02-500)

38. Penicillin/Streptomycin (Thermo Fisher Scientific, catalog number: 15140163)

39. Trypsin-EDTA 0.25% (Thermo Fisher Scientific, catalog number: 25200114)

40. Opti-MEM^TM^ I reduced serum medium (Thermo Fisher Scientific, catalog number: 31985088)

41. Lipofectamine^TM^ 2000 transfection reagent (Invitrogen, catalog number: 11668-019)

42. Clozapine N-oxide (Tocris Bioscience, catalog number: 4936)

43. Bovine plasma (Pel-Freez, catalog number: 37140-1)

44. Gelatin Sepharose 4B (Amersham, catalog number: 17-0956-01)

45. Fibronectin bovine protein, plasma (ThermoFisher, catalog number: 33010018)

46. Sodium acetate (J.T. Baker, catalog number: 3470-01)

47. Acetic acid, glacial (Millipore, catalog number: AX0073-9)

48. PMSF (MilliporeSigma, catalog number: 10837091001)

49. 0.5 M EDTA pH 8.0 (Invitrogen, catalog number: AM9262)

50. CAPS (Fisher Scientific, catalog number: BP321-100)

51. Urea (Sigma, catalog number: U5378-1KG)

52. M199 media (Invitrogen, catalog number: 11150-059)

53. Bovine hypothalamus (Pel-Freez, catalog number: 57117-2)

54. Streptomycin sulfate (Thermo Fisher Scientific, catalog number: 11860038)

55. Endothelial cell growth supplement (Yale Vascular Biology & Therapeutics Program, ECGS)

56. Heparin sodium salt from porcine intestinal mucosa (Sigma, catalog number: H3149)

57. Hexadimethrine bromide (Polybrene) (Sigma, catalog number: H9268-5G)

58. Gibco^TM^ PBS, pH 7.4 (Thermo Fisher Scientific, catalog number: 10-010-031)

59. 10× TBS (Bio-Rad, catalog number: 1706435)

60. 10× Tris/Glycine/SDS (Bio-Rad, catalog number: 1610772)

61. 10× Tris/Glycine (Bio-Rad, catalog number: 1610734)

62. Tween 20 (Sigma, catalog number: P9416)

63. Sodium dodecyl sulfate (AmericanBio, catalog number: AB01920)

64. 2-Mercaptoethanol (Sigma, catalog number: M3148)

65. Bromophenol blue (Sigma, catalog number: B0126-25G)

66. Ammonium persulfate (Sigma, catalog number: 911127-100G)

67. 30% Acrylamide/Bis Solution 37.5:1 (Bio-Rad, catalog number: 1610158)

68. TEMED (Bio-Rad, catalog number: 1610801)

69. Non-fat dry milk Omniblok (AmericanBio, catalog number: AB10109)

70. GLU-GLU monoclonal antibody (BioLegend, catalog number: MMS-115R)

71. GST antibody (Cell Signaling, catalog number: 2625)

72. Tubulin antibody (Invitrogen, catalog number: 62204)

73. Actin antibody (Santa Cruz, catalog number: sc-8432)

74. Reblot Plus Strong antibody stripping solution (10×) (Millipore, catalog number: 2504)

**Solutions**

1. Terrific broth (see Recipes)

2. Bacteria lysis buffer (see Recipes)

3. Glutathione beads wash buffer (see Recipes)

4. Glutathione S transferase elution buffer (see Recipes)

5. Lenti-X 293T cell culturing medium (see Recipes)

6. Cell lysis buffer (see Recipes)

7. 2× SDS buffer (see Recipes)

8. (Optional) Fibronectin (1 mg/mL) from frozen bovine plasma (see Recipes)

**Recipes**

**1. Terrific broth**

Combine 3.6 g of tryptone, 7.2 g of yeast extract, and 1.2 mL of glycerol in a glass flask. Add water to reach a total volume of 270 mL and then sterilize the mixture by autoclaving. Separately dissolve 0.231 g of KH_2_PO_4_ (final concentration 0.017 M) and 1.254 g of K_2_HPO_4_ (final concentration 0.072 M) in water up to 30 mL. Filter this solution through a 0.45 μm filter, mix it with the autoclaved broth, and adjust the volume to 300 mL.

**Table:** 

Reagent	Final concentration	Quantity or volume
Tryptone		3.6 g
Yeast extract		7.2 g
Glycerol		1.2 mL
Water		270 mL
Autoclave the solution above		
KH_2_PO_4_	0.017 M	0.231 g
K_2_HPO_4_	0.072 M	1.254 g
Water		30 mL
Filter 30 mL of phosphate solution through a 0.45 μm filter and add it to the autoclaved broth		
Total		300 mL

**2. Bacteria lysis buffer**

Prepare this buffer fresh each time for optimal effectiveness. Mix 400 μL of 1 M Tris-HCl (pH 7.5) (final 10 mM), 1.2 mL of 5 M NaCl (final 150 mM), 400 μL of Triton X-100 (final 1%), 200 μL of 1 M DTT (5 mM), 40 mg of Lysozyme (1 mg/mL), and protease inhibitor in distilled water using 50 mL conical tubes (40 mL in two tubes). Adjust the solution to 40 mL with distilled water. Mix everything well and keep the buffer on ice until use.

*Note: Avoid EDTA since it interferes with heterotrimeric G protein function.*

**Table:** 

Reagent	Final concentration	Quantity or volume
1 M Tris-HCl (pH 7.5)	10 mM	400 μL × 2
5 M NaCl	150 mM	1.2 mL × 2
Triton X-100	1%	400 μL × 2
1 M DTT	5 mM	200 μL × 2
Lysozyme	1 mg/mL	40 mg × 2
Protease inhibitor		1 capsule per 10 mL
Water		Up to 40 mL × 2

**3. Glutathione beads wash buffer**

Prepare this buffer fresh each time for optimal effectiveness. Mix 400 μL of 1 M Tris-HCl (pH 7.5) (final 10 mM), 1.2 mL of 5 M NaCl (final 150 mM), 400 μL of Triton X-100 (final 1%), and 200 μL of 1 M DTT (5 mM) in distilled water using 50 mL conical tubes (40 mL in two tubes). Adjust the solution to 40 mL with distilled water. Mix everything well and store the buffer on ice until use. Add protease and phosphatase inhibitor prior to usage.

*Note: Avoid EDTA since it interferes with heterotrimeric G protein function.*

**Table:** 

Reagent	Final concentration	Quantity or volume
1 M Tris-HCl (pH 7.5)	10 mM	400 μL × 2
5 M NaCl	150 mM	1.2 mL × 2
Triton X-100	1%	400 μL × 2
1 M DTT	5 mM	200 μL × 2
Water		Up to 40 mL × 2

**4. Glutathione S transferase elution buffer**

Mix 2 mL of 1 M Tris-HCl (pH 7.5) (final 10 mM), 6 mL of 5 M NaCl (final 150 mM), 2 mL of Triton X-100 (final 1%), and 1 mL of 1 M DTT (5 mM) in distilled water using a 200 mL glass bottle. Adjust the solution to 50 mL with distilled water. Mix everything well and adjust the pH to 7.2 using 10 N NaOH. Make up the final volume with distilled water to 200 mL. Store at 4 °C until use. Add protease and phosphatase inhibitor just before use.

**Table:** 

Reagent	Final concentration	Quantity or volume
1 M Tris-HCl (pH 7.5)	10 mM	2 mL
5 M NaCl	150 mM	6 mL
Triton X-100	1%	2 mL
1 M DTT	5 mM	1 mL
Glutathione	50 mM	3.07 g
10 N NaOH		To pH 7.2
Water		Up to 200 mL
Store at 4 °C and aliquot in 10 mL before use		
Protease and phosphatase inhibitor	1×	100 μL

**5. Lenti-X 293T cell culturing medium**

Remove 55 mL from the DMEM bottle to make the total volume 500 mL. Pour in 50 mL of FBS and 5 mL of penicillin/streptomycin. Mix gently to combine everything.

**Table:** 

Reagent	Final concentration	Quantity or volume
DMEM		445 mL
FBS	10%	50 mL
Penicillin/Streptomycin		5 mL
Total		500 mL

**6. Cell lysis buffer**

Prepare this buffer fresh each time for optimal effectiveness. Mix 100 μL of 1 M Tris-HCl (pH 7.5) (final 10 mM), 300 μL of 5 M NaCl (final 150 mM), 100 μL of Triton X-100 (final 1%), 50 μL of 1 M DTT (5 mM), and protease and phosphatase inhibitor in distilled water in 15 mL conical tubes. Add 20 μg of GST-GINIP protein for Gi assay or 20 μg of GST-GRK2 N-terminal domain for Gq assay. Adjust the solution to 10 mL with distilled water. Mix everything well and store on ice until use.

*Note: Avoid EDTA since it interferes with heterotrimeric G protein function.*

**Table:** 

Reagent	Final concentration	Quantity or volume
1 M Tris-HCl (pH 7.5)	10 mM	100 μL
5 M NaCl	150 mM	300 μL
Triton X-100	1%	100 μL
1 M DTT	5 mM	50 μL
GST-bait protein (GINIP for Gi, GRK2 N for Gq)	2 μg/mL	1 aliquot (20 μg)
Protease and phosphatase inhibitor		
Water		Up to 10 mL

**7. 2× SDS buffer**

Mix 3 mL of 1 M Tris-HCl (pH 6.8), 5 mL of glycerol, 10 mL of 10% SDS, and 2 mL of beta-mercaptoethanol. Take a small pinch of bromophenol blue in 1.5 mL tubes and add distilled water to make a suspension. Take water-saturated bromophenol blue into the mixture. Adjust the total volume to 20 mL using distilled water.

**Table:** 

Reagent	Final concentration	Quantity or volume
1 M Tris-HCl (pH 6.8)	0.12 M	3 mL
Glycerol	20%	5 mL
10% SDS	4%	10 mL
Beta-mercaptoethanol		2.5 mL
Water-saturated bromophenol blue		2 mL
Water		Up to 25 mL

**8. (Optional) Fibronectin (1 mg/mL) from frozen bovine plasma**

Prepare a gelatin Sepharose 4B column by pouring 25 mL of resin into a column, and wash sequentially with cleaning buffers (cleaning buffer #1: 16× of column volume, and cleaning buffer #2: 16× of column volume) without allowing it to dry. Fresh frozen bovine plasma is then thawed, volume-measured, treated with 1 mM PMSF, and centrifuged at 20,000× *g* to remove precipitates. The supernatant is carefully loaded onto the prepared column without disturbing the column bed.

The column undergoes subsequent washes (12 column volumes of column wash buffer #1, 4 column volumes of column wash buffer #2, and 2 column volumes of column wash buffer #1); then, elute fibronectin with 50 mL of elution buffer. Collect the eluate in 1.5 mL fractions and measure optical density at 280 nm to determine fibronectin concentration, combining peak fractions from an O.D. starting at 0.7. Dialyze overnight at 4 °C in dialysis buffer, with a change of buffer the next morning with additional incubation for 3 h. Measure the optical density again, dilute the fibronectin to 1 mg/mL (OD_280_ of 1.3 equals 1 mg/mL of fibronectin), aliquot, freeze in liquid nitrogen, and store at -80 °C.

**8.1. Cleaning buffer #1 (200 mL)**

**Table:** 

Reagent	Final concentration	Volume
1 M Tris-HCl (pH 8.5)	100 mM	20 mL
5 M NaCl	500 mM	20 mL
Water		Up to 200 mL

**8.2. Cleaning buffer #2 (200 mL)**

**Table:** 

Reagent	Final concentration	Volume
Sodium acetate	100 mM	
5 M NaCl	500 mM	20 mL
Acetic acid		Adjusting to pH 4.5
Water		Up to 200 mL

**8.3. Column wash buffer #1 (1 L)**

**Table:** 

Reagent	Final concentration	Volume
PBS	1×	Up to 1 L
1 M PMSF	1 mM	1 mL
0.5 M EDTA	2 mM	4 mL

**8.4. Column wash buffer #2 (1 L)**

**Table:** 

Reagent	Final concentration	Quantity or volume
PBS	1×	Up to 1 L
1 M PMSF	1 mM	1 mL
NaCl	1 M	58.44 g
0.5 M EDTA	2 mM	4 mL

**8.5. Dialysis buffer (4 L)**

**Table:** 

Reagent	Final concentration	Quantity or volume
CAPS	10 mM	8.85 g
1 M PMSF	1 mM	4 mL
5 M NaCl	0.15 M	120 mL
0.5 M EDTA	2 mM	8 mL
10 N NaOH		Adjust to pH 11.0
Water		Up to 4 L

**8.6. Elution buffer (100 mL)**

**Table:** 

Reagent	Final concentration	Quantity or volume
Dialysis buffer	1×	Up to 100 mL
Urea	4M	24 g

*Note: Commercially available fibronectin (e.g., ThermoFisher, catalog number: 33010018) can also be used.*

**Laboratory supplies**

1. 14 mL round-bottom high-clarity PP test tube (BD, Falcon, catalog number: 352059)

2. 15 mL conical tube (Corning, catalog number: 430791)

3. 50 mL conical tube (Corning, catalog number: 430828)

4. 10 cm bacteriological Petri dish (Corning, catalog number: 351029)

5. Zeba^TM^ spin desalting columns, 7 K MWCO, 10 mL (Thermo Fisher, catalog number: 89893)

6. Corning^®^ 100 mm TC-treated culture dish (Fisher Scientific, catalog number: 08-772-22)

7. 25 mL pipettes (Greiner, catalog number: 760160)

8. 10 mL pipettes (Greiner, catalog number: 607180)

9. 5 mL pipettes (Greiner catalog number: 606180)

10. TipOne^®^ RPT pipette tip refills (1,250 μL) (USA Scientific, catalog number: 1161-1720)

11. TipOne^®^ 200 μL natural pipette tips in racks (USA Scientific, catalog number: 1111-0840)

12. TipOne^®^ filter tips 10 μL (USA Scientific, catalog number: 1121-3810)

13. Fisherbrand^TM^ Premium microcentrifuge tubes, 1.5 mL (Fisher Scientific, catalog number: 05-408-129)

14. Tube Posi-Click 1.7 mL natural graduated (labForce, catalog number: 1149K01)

15. 0.45 μm Millex^TM^-HP filter unit (sterile) (Millipore, catalog number: SLHP033RS)

16. 10 mL syringes (Fisher Scientific, catalog number: 14-823-2A)

17. Nitrocellulose membrane (Bio-Rad, catalog number: 10484059)

18. Filter paper

## Equipment

1. pH meter (METTLER TOLEDO, model: SevenDirect SD20)

2. Scales (METTLER TOLEDO, model: ML802T and XSR105)

3. Magnetic stirrer (Corning, model: PC351)

4. Laboratory centrifuge with rotors for 15 and 50 mL conical tubes (Beckman Coulter, model: Avanti JXN-26)

5. Autoclave (Steris, model: AMSCO 250LS)

6. Forced-air incubator (for bacteria plates) (Yamato, model: IC400)

7. Bacterial incubator shaker (New Brunswick, model: Innova 44/44R or Innova 4000)

8. Sonic dismembrator (Fisher Scientific, model: Model 500)

9. Laboratory centrifuge with rotors for 1.5 mL microcentrifuge tubes (Thermo Scientific, model: Sorvall^TM^ Legend^TM^ Micro 21 Microcentrifuge)

10. Cell incubator (SANYO, model: MCO-19AIC)

11. Biological safety cabinet (LabGard, model: NU-425-600)

12. Chemiluminescence imaging system (Syngene, G:BOX, model: Chemi-XX9)

13. Racks

14. Liquid nitrogen (N_2_) tank

15. Freezer (-80 °C)

16. Freezer (-20 ° C)

17. Refrigerator (4°C)

## Software and datasets

1. GeneSys (Syngene, Version 1.8.12.0, Database Version 2.2)

2. ImageJ/Fiji

3. Microsoft Excel

4. GraphPad Prism

## Procedure

**A. Bacteria transformation with Gα protein effector constructs**

1. (Optional) Plasmid amplification

a. Transform Stbl3 *E. coli* cells with the constructs expressing either GST-tagged GINIP or GST-tagged GRK2 N-terminal domain and spread the transformed bacteria suspension onto an LB agar plate containing 100 μg/mL ampicillin. Follow a standard transformation protocol to achieve high-efficiency uptake of the plasmid DNA into the *E. coli* cells.

b. Pick a well-isolated single colony and inoculate it into the appropriate volume of LB broth containing 100 μg/mL ampicillin. Incubate the culture overnight at 37 °C with shaking.

c. Collect the plasmid using a standard plasmid isolation kit following the manufacturer’s protocol.

2. Transformation of bacteria for protein expression

a. Transform BL21 (DE3) *E. coli* cells with the plasmid encoding GST-bait protein. Follow a standard transformation protocol from the manufacturer.

3. Culturing bacteria and inducing protein expression

a. Inoculate transformed *E. coli* in several tubes of 3 mL Terrific broth with 100 μg/mL ampicillin. Incubate the cultures overnight at 37 °C with shaking.

b. Add 100 μg/mL ampicillin into 300 mL of Terrific broth prepared in a glass flask as described in the Recipes section. Add 10 mL of bacterial preculture into 300 mL of Terrific broth.

c. Grow the culture at 37 °C for 3 h.

d. Induce protein expression by adding IPTG (stock 1 M at -20 °C) to a final concentration of 100 μM.

e. Incubate cells at 30 °C for 5 h with strong agitation.

**B. Collection and purification of GST-tagged bait protein**

1. Harvesting cells

a. Collect the cells by centrifugation at 6,000× *g* for 15 min at 4 °C.

b. Immediately freeze the pellet using liquid nitrogen.

*Note: After freezing, the bacterial pellet can be stored at -80 °C for future use.*

2. Protein purification

a. Thoroughly resuspend the cell pellet in bacterial lysis buffer on ice, using 10 mL of buffer per 50 mL of bacterial culture.

b. Incubate the suspension on ice for 15 min.

c. Lyse the cells by sonication: perform three cycles, each for 30 s at 50% duty cycle at 50% output, with a 30 s break between cycles. Maintain the samples on ice to prevent heat-induced protein degradation.

d. Centrifuge the lysate at 20,000× *g* for 30 min at 4 °C.

e. While centrifuging the bacterial lysate, wash 2 mL of glutathione Sepharose 4B beads with 40 mL of glutathione beads wash buffer for equilibration.

f. Add the supernatant from the centrifugation to the equilibrated glutathione Sepharose 4B beads.

g. Rotate the mixture at 4 °C for 1 h for optimal binding of GST-tagged proteins to the beads.

h. Centrifuge the sample at 500× *g* for 30 min at 4 °C.

i. Remove the supernatant, resuspend beads in 40 mL of glutathione beads wash buffer, and centrifuge again.

j. Repeat the wash steps in B2i three times more (a total of four washes).

k. Elute the bound GST-tagged proteins with 3 mL of glutathione S transferase elution buffer. Gently rotate overnight at 4 °C.

l. Centrifuge the eluted sample at 500× *g* for 30 min at 4 °C.

m. Collect the supernatant and desalt the eluted proteins on Zeba^TM^ spin desalting columns, 7 K MWCO, 10 mL to remove excess glutathione and perform buffer exchange.

n. Measure protein concentration using the Bio-Rad Protein Assay Dye Reagent Concentrate according to the manufacturer’s protocol.

o. Aliquot the proteins into 20 μg aliquots, freeze them using liquid nitrogen, and store at -80 °C. Thaw aliquots immediately before use.

**C. Packaging lentivirus for GluGlu-tagged Gα protein using Lenti-X 293T cells**

*Note: If your cells of interest are amenable to transfection, you can skip this section and proceed to Section D.*

1. Culture preparation

a. Maintain Lenti-X 293T cells in Lenti-X 293T culturing medium at 37 °C with 5% CO_2_.

2. Transfection for lentivirus production

a. Ensure cells are at approximately 70%–80% confluence on a 100 mm dish in antibiotic-free DMEM supplemented with 10% FBS on the day of transfection.

b. Prepare DNA mixture in 500 μL of Opti-MEM including:

• 5 μg of viral vector plasmid encoding GluGlu-tagged Gi mutant, or both GluGlu-tagged Gq mutant and Ric8A

• 5 μg of packaging plasmid (psPAX2)

• 5 μg of envelope plasmid (pMD2.G)

c. Use 25 μL of Lipofectamine^TM^ 2000 transfection reagent mixed in 500 μL of Opti-MEM.

d. Combine the DNA mixture and Lipofectamine 2000 suspension completely.

e. Wash Lenti-X 293T cells with Opti-MEM and add 4 mL of Opti-MEM to the culture.

f. Incubate the mixture for 20 min, add to the cells, and incubate for 2 h.

g. Replace the Opti-MEM medium with 10 mL of HUVEC culturing medium lacking penicillin/streptomycin.

3. Harvesting lentivirus

a. Incubate transfected cells at 37 °C with 5% CO_2_ for 24 h.

b. Collect the supernatant containing lentivirus particles and centrifuge at 300× *g* for 5 min to remove floating cells.

c. Filter the supernatant through a 0.45 μm filter to remove remaining cell debris.

d. (Optional) Concentrate the lentivirus by ultracentrifugation or using a PEG-it virus precipitation solution according to the manufacturer’s instructions.

**D. Preparation of cells expressing GluGlu-tagged Gα protein**

*Note: Depending on whether cells are amenable to transfection, select either transfection (D1) or lentivirus infection (D2).*

1. Transfecting cells with a plasmid encoding GluGlu-tagged Gi or GluGlu-tagged Gq protein plus Ric8A

a. Make sure that cells are healthy and at 50%–80% confluence in a 100 mm dish in antibiotic-free medium.

b. Prepare a DNA mixture in 500 μL of Opti-MEM including:

• 5 μg of viral vector plasmid encoding GluGlu-tagged Gi mutant, or both GluGlu-tagged Gq mutant and Ric8A

c. Use 25 μL of Lipofectamine^TM^ 2000 transfection reagent mixed in 500 μL of Opti-MEM.

d. Combine the DNA mixture and Lipofectamine 2000 suspension and incubate for 20 min to allow for complex formation.

e. Wash the cells with 4 mL of Opti-MEM.

f. Add the mixture to the cells and incubate for 2 h.

g. Exchange medium with the appropriate antibiotic-free medium and incubate cells for 24–48 h until the cells express GluGlu-tagged G protein.

2. Infecting cells with lentivirus encoding GluGlu-tagged Gi or GluGlu-tagged Gq protein plus Ric8A

a. Maintain cell lines or primary cells in their optimal medium. As for HUVECs, confirm early passage cells (<3) are cultured on a dish coated with 10 μg/mL fibronectin in HUVEC culturing medium [16].

b. Place cells in the appropriate growth medium until they reach approximately 70%–80% confluence.

c. Add lentivirus to the culture medium with polybrene (stock: 10 mg/mL, working concentration: 10 μg/mL) to enhance infection efficiency. Typically, we avoid virus freezing and add a 1:2–1:10 dilution of virus-containing medium from Section C to HUVECs.

d. Incubate the cells with the virus overnight at 37 °C.

e. Replace the virus-containing medium with fresh growth medium 24 h post-infection.

f. Incubate the cells for 3 days until GluGlu-tagged Gα protein is well expressed.

**E. Pull-down assay**

*Note: Heterotrimeric Gα protein exhibits GTPase activity, leading to a gradual decrease of active Gα protein in cell lysate. Maintain the samples under cold conditions and complete the entire process as quickly as possible, from starting cell lysis to finishing pulldown.*

1. Cell lysis

a. Stimulate HUVECs or cells of interest with desired GPCR ligands or other stimuli.

b. Wash cells quickly with cold PBS once.

c. Extract cells with cell lysis buffer (200 μL of cell lysis buffer per well of a 6-well plate) supplemented with 2 μg/mL of GST-GINIP for GluGlu-tagged Gi or GST-GRK2N for GluGlu-tagged Gq. Add lysis buffer directly to the plates and immediately proceed with the pulldown step.

d. Reserve 10 μL of cell lysate on ice as input for western blotting.

2. Pulldown

a. Pre-equilibrate glutathione Sepharose 4B beads with glutathione bead wash buffer and prepare 50% bead slurry before cell lysis.

b. Add 50 μL of the glutathione Sepharose 4B bead slurry to the lysate.

c. Gently rotate the mixture for 10 min at 4 °C.

d. Centrifuge at 500× *g* for 1 min.

e. Wash beads with 900 μL of cold cell lysis buffer three times.

3. Elution and western blot analysis

a. Solubilize the proteins bound to beads by adding 30 μL of 2× SDS buffer. Similarly, add 10 μL of 2× SDS buffer to the reserved cell lysate.

b. Incubate the samples at >95 °C or in boiling water for 5 min.

*Note: Once boiled, samples can be stored at -20 °C for future use.*

c. Load 8 μL of the solubilized protein samples (both pulldown and lysate) onto an SDS-PAGE gel. Run the gel according to standard protocols.

*Note: 10% acrylamide gel is optimal to observe Gα proteins.*

**Figure 2.**
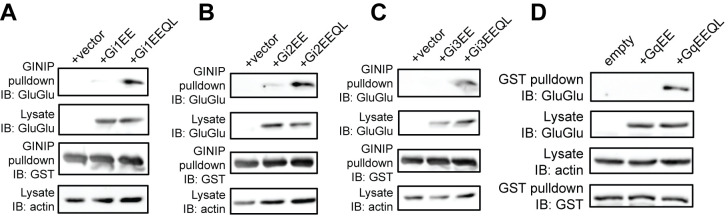
Validation of Gi protein pull-down assay and Gq protein pull-down assay using GTPase-deficient mutants. (A) Gi1 activation, (B) Gi2 activation, (C) Gi3 activation, (D) Gq activation. Figure is from Tanaka et al. [16].

**Figure 3.**
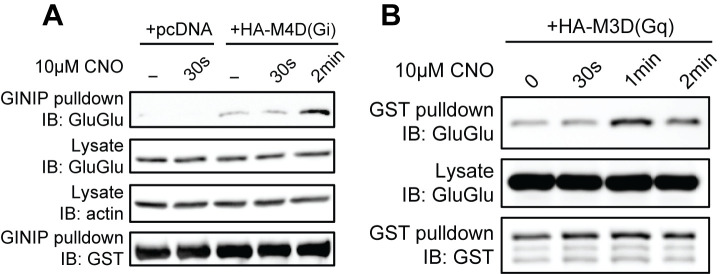
Validation of Gi and Gq protein pull-down assays using artificial ligand-activated DREADD G-protein coupled receptors (GPCRs). (A) Activation of Gi, (B) activation of Gq. Figure is from Tanaka et al. [16].

**Figure 4.**
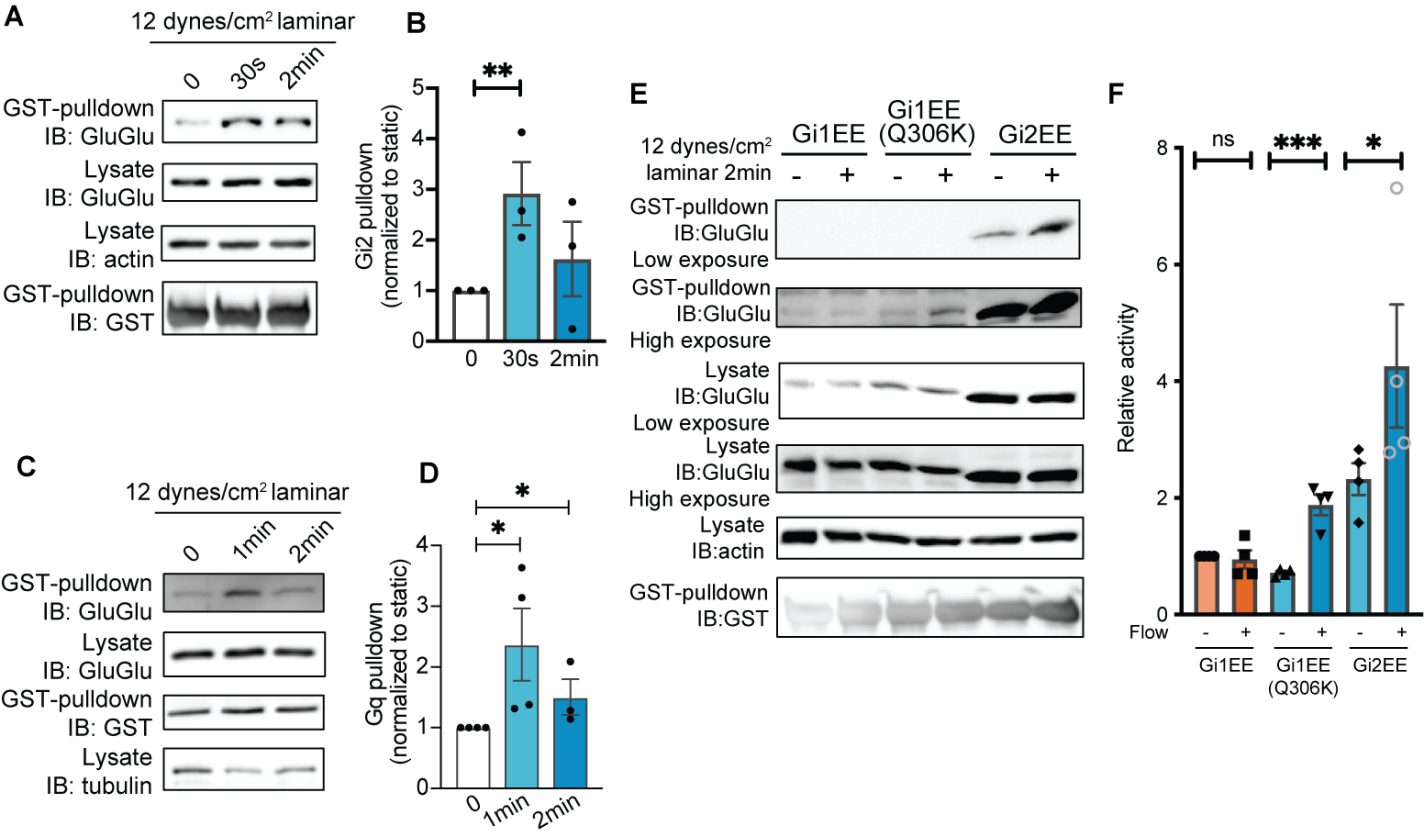
Visualization of signaling differences among Gα isoforms in endothelial cells under fluid shear stress. (A) GINIP pull-down assay for activation of Gi2 by fluid shear stress. (B) Quantification of (A). **P = 0.0185; *Student*’s t test. (C) GRK2N pull-down assay for activation of Gq. (D) Quantification of (C). *P < 0.05; *Student*’s t test. (E) GINIP pull-down assay could visualize the activation differences between Gi1 isoform, Gi1 gain-of-function mutant, and Gi2 isoform. (F) Quantification of (E). *P = 0.0304, ***P = 0.0017; *Student*’s t test. All error bars are SEM. Figure is from Tanaka et al. [16].

d. Transfer the proteins from the gel to a nitrocellulose membrane.

e. Block the membrane with 5% Non-fat dry milk Omniblok for 1 h at room temperature.

f. Incubate the membrane with primary antibody against the GluGlu epitope at a 1:1,000 dilution at 4 °C overnight with gentle shaking.

g. Wash the membrane three times with TBS-T (1× TBS with 0.1% Tween-20).

h. Incubate the membrane with HRP-conjugated secondary antibody at room temperature for 1 h with gentle shaking.

i. Wash 4× with TBS-T.

j. Use an enhanced chemiluminescence (ECL) substrate to visualize the bands corresponding to the GluGlu-tagged G protein pulldowns with GST-bait protein.

k. Incubate the membrane with Reblot Plus Strong antibody stripping solution for 15 min and then wash the membrane twice with TBS-T.

l. Cut the membrane between the pulldown and lysate lanes. Re-blot the pulldown lanes with a GST antibody (1:1,000 dilution) and the lysate lanes with a loading control, such as tubulin (1:10,000 dilution) or actin (1:2,000 dilution) (**Figures 2–4**).

## Data analysis

To quantify gel images using ImageJ, first convert to 8-bit grayscale via *Image > Type > 8-bit* for consistent data analysis across different computer or machine settings. Use the rectangular selection tool to outline the band in the first lane. Navigate to *Analyze > Gel* and select *Select First Lane*. Move the selection rectangle to the next lane and continue selecting remaining lanes with *Analyze > Gel > Select Next Lane* until all are outlined, then choose *Analyze > Gel > Plot Lanes* to generate intensity profiles for each lane.

Analyze the generated intensity plot curves by manually drawing lines to surround the area under the curve. Once drawn, click on the area under the curve with the *Wand Tool* to obtain areas as band intensities. Finally, export the quantified results using the *File > Save As* feature or by simply copying the resultant window data for further analysis in Microsoft Excel or GraphPad Prism. Analyze statistics from multiple repeats as appropriate for the experimental design (**Figure 4**).

## Validation of protocol

This protocol has been used and validated in the following article:

• Tanaka et al. [16]. Latrophilin-2 mediates fluid shear stress mechanotransduction at endothelial junctions (Figures 1 and 2, Appendix Figure S3).

## General notes and troubleshooting

**General notes**

1. The process from cell lysis to pulldown should be performed as quickly as possible.

2. Include the GST-bait protein in the lysis buffer. This procedure facilitates the binding between Gα protein and effector protein as cells lyse, which also improves signal intensity.

3. Avoid EDTA in all buffers and reagents, since EDTA affects Gα protein activity.

**Troubleshooting**

Problem 1: Cells exhibit poor health following virus infection.

Possible cause: Excessive viral load.

Solution: Decrease the amount of virus applied.

Problem 2: Cells do not have sufficient expression of GluGlu-tagged Gα protein.

Possible cause: 1) Insufficient viral load. 2) Target cells are difficult to transduce.

Solution: 1) Increase the virus amount and avoid virus freezing. 2) Establish a plasmid with a drug selection marker or a sorting marker. Plasmid sequences can be found in File S1.

## Supplementary information

The following supporting information can be downloaded here:

1. File S1. GluGlu-tagged Gα plasmid sequences.
